# Integrated Programs for Early Recognition of Severe Mental Disorders: Recommendations From an Italian Multicenter Project

**DOI:** 10.3389/fpsyt.2019.00844

**Published:** 2019-11-15

**Authors:** Alberto Parabiaghi, Linda Confalonieri, Nadia Magnani, Antonio Lora, Emanuela Butteri, Katia Prato, Marco Vaggi, Mauro Emilio Percudani

**Affiliations:** ^1^Department of Neuroscience, Mario Negri Institute for Pharmacological Research, Milan, Italy; ^2^Department of Mental Health and Addiction Services, ASST Grande Ospedale Metropolitano “Niguarda”, Milan, Italy; ^3^Department of Mental Health, Azienda USL Toscana Sud Est, Grosseto, Italy; ^4^Department of Mental Health and Addiction Services, ASST Lecco, Lecco, Italy; ^5^Department of Mental Health and Addiction Services, ASST Fatebenefratelli-Sacco, Milan, Italy; ^6^Department of Mental Health and Addiction Services, ASST Rhodense, Milan, Italy; ^7^Department of Mental Health and Addiction Services, ASL 3 Genova, Genova, Italy

**Keywords:** at-risk mental state, psychosis, ultra high risk, early intervention, transition, prevention strategies, community coalitions

## Abstract

The onset of mental disorders often occurs in adolescence or young adulthood, but the process of early diagnosis and access to timely effective and appropriate services can still be a challenge. The goal of this paper is to describe a pilot case of implementation of the ultra-high-risk (UHR) paradigm in six Italian departments of mental health employing an integrated approach to address clinical practice and service organization for youth in a broader preventive perspective. This approach entailed the integration of the UHR paradigm with a service provision model which prioritizes prevention and the promotion of local community coalitions to improve youth service accessibility. The multicenter Italian project “Integrated programs for recognition and early treatment of severe mental disorders in youths” funded by the National Centre for Disease Prevention and Control (CCM2013 Project) implemented in three Italian regions will be described. As a result of synergic actions targeting accessibility of young individuals to innovative youth mental health teams, a total of 376 subjects aged 15–24 years were recruited by integrated youth services within 12 months. Subjects have been screened by integrated multidisciplinary mental health youth teams employing standardized procedure and evidence-based clinical assessment instruments for at-risk mental states in young subjects [e.g., Comprehensive Assessment of At-Risk Mental States (CAARMS)]. Considering the three UHR categories included in CAARMS, the percentage of UHR subjects was 35% (n = 127) of the sample. In conclusion, future strategies to improve the organization of youth mental health services from a wider preventive perspective will be proposed.

## Critical Issues in Implementing the “Ultra-High-Risk” and the “Transition” Paradigm in Routine Clinical Practice

Over the last 25 years, a significant number of studies focused on the early detection and intervention for severe mental disorders. The onset of psychosis often occurs in adolescence or young adulthood, and the process of early diagnosis and access to timely effective interventions is still a challenge for mental health services. In the last decades, several clinical criteria have been identified with the aim to detect in youths the occurrence of a clinical high-risk state for psychosis as possible prodromal phase of psychotic disorders, including several labels as ultra-high-risk (UHR), clinical high risks (CHR), or at-risk mental states (ARMS) (1).

The UHR and transition paradigm involves specific criteria (extensively applied in numerous countries) to diagnose the UHR in help-seeking individuals (2–4). Inclusion requires the presence of one or more of the following: attenuated psychotic symptoms (APSs), brief limited intermittent psychotic episodes (BLIPs), trait vulnerability, schizotypal personality disorder plus a marked decline in psychosocial functioning [genetic risk and deterioration syndrome (GRD) and unspecified prodromal symptoms (UPSs)] (1).

The basic assumption for the UHR and transition paradigm is that it is possible to identify people who are at risk and in need of preventive interventions by applying a binary diagnostic category (psychosis risk vs. no psychosis risk) in young help-seeking individuals. In those subjects, the transition to psychotic disorders is clinically significant, with studies showing transition rates of 15–20% after the first year, and of 30% after 3 years (4). Consequently, according to the “Clinical Stage Model” (5), evidence-based interventions aimed at promoting the recovery process targeting a specific stage of the disorder (e.g., APSs) and to prevent the progression towards the following stages (e.g., first psychotic episode) should be provided (6–8).

Recently, alternative transdiagnostic perspectives have been presented (9–12). The concept of “at-risk mental states” can be conceived as a cue of wider and more general transdiagnostic psychological distress and vulnerability and psychotic experiences observed in youth as markers of the severity of multidimensional psychopathology rather than a binary psychosis risk criterion (9, 10). Epidemiological studies, in fact, showed that APSs as well as psychotic experiences are closely associated with non-psychotic disorders and/or sub-diagnostic non-psychotic psychopathology (13, 14).

An updated view for planning youth mental health services should embrace a wider perspective focusing the full range of person-specific psychopathology (9) in young subjects with emotional distress. Therefore, widening the concept of “at-risk mental state” with the aim of identifying risk and protective factors for youth mental health, and promoting access of young subjects to mental health services could be the line of action. Accordingly, beyond assessing the binary risk of psychosis, young individuals should be viewed as targets for wider secondary prevention strategies, also aimed at reducing stigma and improving access to innovative youth mental health services.

In Italy, adult and child–adolescent mental health services are strictly age-based and show a low level of integration. The activities of both services focus on patients outside the critical age range of 14–25. Child–adolescent mental services employ the majority of their resources on patients with neurodevelopment disorders and learning disabilities, whereas adult mental health services are much involved in the treatment and rehabilitation of severe and persistent mental disorders. In terms of prevention, a number of projects in the area of early detection and intervention of the psychosis onsets have been implemented in Italy (15, 16). From the national survey promoted by the *Associazione Italiana per la Prevenzione delle Psicosi* (16), the national diffusion of the model of Early Intervention in Psychosis can be estimated between 20% and 45%, with a higher diffusion in Northern and Central Italy than in Southern Italy and the Islands. However, all those projects are focused on early detection of psychosis and not on prodromal symptoms and UHR conditions in help-seeking youth.

Despite clear national and regional programmatic indications to improve primary and secondary prevention actions through the identification of at-risk conditions in youth (Mental Health Action Plan 2013–2020, Italian Ministry of Health), the development of multicentric standardized actions focused on UHR assessment as well as to services’ accessibility is still not frequent within the mental health Italian system. Moreover, recent findings (17, 18) show that, in Italy, yearly treated prevalence was the lowest for people aged 18–24 and that accessibility to public mental health services should be increased for people below 30 years in order to improve early-psychosis outcome.

The aim of this paper is to describe a pilot case of implementation of youth mental health services within the Italian framework according to three key elements: 1) enhanced secondary prevention-oriented actions including the screening of at-risk mental states in young subjects through standardized procedures and instruments; 2) higher services’ accessibility of young individuals with sub-threshold symptoms with specific attention to vulnerable or at-risk groups; and 3) the establishment of youth mental health teams with high level of integration between the adult and child–adolescence mental health services.

## The CCM2013 Project: a Pilot Implementation of the High-Risk Paradigm Within Italian Community Services

### Aims

The Italian project “Integrated programs for recognition and early treatment of severe mental disorders in youths” was funded by the National Centre for Disease Prevention and Control (CCM2013 Project). The project aimed at implementing the UHR paradigm in six departments of mental health in Italy (sited in Lombardy, Liguria, and Tuscany) developing an integrated approach to address clinical practice and service organization for youth in a broader preventive perspective. Specifically, the project’s goal was the integration of the traditional UHR paradigm with a service provision model which prioritizes prevention and the promotion of local community coalitions (19). Community coalitions are participatory models of intervention aimed at mobilizing people to promote community health in several domains, including mental health (20). Community prevention coalitions are formal, long-term collaborations composed of diverse non-institutional organizations (e.g., schools, ethnic and religious associations) aiming at developing effective prevention programs to promote adolescent health and well-being (21).

The CCM2013 Project intended to put into practice the national programmatic indications (Mental Health Action Plan 2013–2020, Italian Ministry of Health) to promote the accessibility to services and the preventive screening of young adults and adolescents. Its aims included to ensure effective evidence-based clinical assessment of ARMS in young subjects (15–24 years old) and to encourage youths’ participation in different community organizations through the Community Coalition model (19). Moreover, it aimed at improving the integration between child–adolescent and adult mental health services through the creation of integrated and multidisciplinary youth mental health teams (22).

### Implementation Actions

The implementation of the prevention-oriented model involved specific actions and organizational changes as follows. Multidisciplinary youth mental health teams integrating various child, adolescent, and adult mental health professionals were created (Integrated Youth Teams) thus promoting cooperation between services. All team members (psychiatrists, psychologists, social workers, etc.) were trained in the detection of risk for developing serious mental disorders. New locations have been identified and used in order to offer attractive and low-stigmatizing physical environments to young individuals accessing to the Integrated Youth Teams. Those locations were separated from the routine mental health service sites and hospitals, and possibly located near public areas visited by young individuals (e.g., parks, schools). Attractive signs with creative names indicating those sites were created. Locations were provided with modern non-medical furniture.

Local community coalitions were activated, aiming at promoting early referral to appropriate services and inclusive pathways for young people experiencing mental distress (20–22). Community coalitions involved different stakeholders (associations, schools, religious and ethnic organizations, family doctors and general practitioners, etc.) among which members of community coalitions’ coordination boards were selected. The boards, after receiving specific training sessions on youth mental health, implemented local initiatives aimed at raising awareness on mental health and at reducing stigma in young individuals (e.g., information day inside schools and associations, art exhibitions focused on mental health in youth, online initiatives, etc.). Actions aimed at favoring accessibility for vulnerable youth groups were implemented, e.g., public events in cooperation with ethnic associations aimed at increase awareness on youth mental health. Local coalitions fostered the integration between youth mental health teams and community stakeholders in order to promote rapid and effective referral pathways.

During 12 months, help-seeking subjects aged 15–24 years have been recruited in six mental health departments within three Italian regions. Enrollment criteria for the UHR assessment have been designed in order to include as many young subjects as possible from the age of 15–24 presenting emotional distress and a wide range of psychological difficulties. Cutoff scores of the clinical assessment instruments were not required as inclusion criteria. Exclusion criteria for the UHR assessment were: age not included in the range 15–24 and the presence of mental retardation (IQ score less than 80).

### Procedure and Instruments

Patients’ referral to the project occurred through diversified ways: first, young subjects could be referred from the community coalition stakeholders (including family doctors and pediatricians); second, patients could be referred from other mental health services; third, they could have direct access to the project. Similarly, members of the community coalition boards could discuss referral cases during their meetings in order to identify adequate strategies to support young subjects and to help them contacting the Youth Integrated Teams. After obtaining informed consent, the assessment procedure started within maximum 3 working days.

Subjects have been assessed using a standardized procedure and clinical instruments validated to assess ARMS conditions. The following self-report questionnaires have been used: the Italian version of General Health Questionnaire-12 (GHQ-12), which is a 12-item self-report questionnaire used for identifying minor psychiatric disorders (23), and the Italian version of the Prodromal Questionnaire-16 (PQ-16), which includes 16 self-reported items screening for the risk of psychosis (24, 25). A psychotherapist and/or a psychiatrist (adequately trained to administer the CAARMS instrument) administered the Comprehensive Assessment of At-Risk Mental States (CAARMS) to assess at-risk mental states and prodromal conditions (26). Moreover, Global Assessment of Function (GAF) (27), Social and Occupational Functioning Assessment Scale (SOFAS) (28), and Health of Nation Outcome Scale (HoNOS) and Health of Nation Outcome Scale for Children and Adolscents (HoNOSCA) (29) were used as measures of the health and social functioning of patients.

The assessment regularly involved an integrated multidisciplinary team of mental health professionals, consisting in psychiatrists, psychologist–psychotherapists (with a minimum of 3 years of clinical practice experience and qualified on UHR assessment), social workers, and nurses. Subsequently, clinical meetings between the mental health professionals were routinely scheduled to identify UHR subjects and to define the type of intervention to recommend (e.g., cognitive–behavioral psychotherapy protocols, and/or pharmacological treatments, social skills training, etc.). However, the project’s main focus was at the screening level of UHR conditions; in fact, the project’s aims did not involve any monitoring of treatment outcome for empirical research purpose.

After the assessment, subjects could be referred to different patterns of care, from the Integrated Youth Team, to the activation of specific supportive interventions with the community coalition board or the routine clinical treatment at standard mental health services (e.g., young patients that not satisfied the UHR criteria). Interventions were provided within maximum 2 weeks without any waiting list, according to the Clinical Stage Model (5) and prioritizing psychotic onsets.

Regarding the control quality of the organizational setup, the whole implementation process was monthly monitored by each center. Progresses were registered into the project monitoring forms and reported to the funding authorities each semester. Reports included information regarding actions implemented to promote the accessibility and attractiveness of locations, number and contents of Integrated Youth Team clinical review meetings, etc. In terms of quality control of the community coalitions’ actions, projects’ inspectors monitored through semestral reports and in-site visits the number and contents of community coalition board meetings, types and number of events organized, and procedures implemented to increase awareness and promote accessibility of vulnerable youth groups.

Each semester, monitoring visits were performed to verify and ensure that all the centers were implementing coordinated actions consistent with projects’ aims. Continuous training and group supervision meetings (every 2 months) with youth mental health experts were organized in order to guarantee the quality of the clinical assessments.

### Results

The proposed prevention-oriented clinical model was developed in all participating centers. This implied a change in the way young patients were identified and assessed. Moreover, through the promotion of local community coalitions, an innovation in the implementation of prevention-oriented programs was introduced. In fact, in all participating centers, local community coalitions were promoted. All community boards spontaneously performed at least one local action in the field of mental health promotion and/or mental health stigma prevention. They took an active role in connecting vulnerable youths with formal and informal help resources by activating and supporting their social networks and families.

The main results of this project consisted in the implementation of the actions described in the previous section. In order to provide a figure of the recruitment and UHR assessment phase, concise data will be presented. During the recruitment phase (from April 2015 to April 2016), 376 participants were referred. Twelve of them dropped out as they did not show up after first contact. Finally, 364 subjects were fully assessed. Participants were equally distributed in terms of gender (53% females). With respect to the age range (mean = 19.3; SD: ± 2.5), 29% of the patients (n = 105) were 15–17 years old, 48% (n = 175) were 18–21 years old, and 23% (n = 84) were 22–24 years old. Using the criteria defined by the CAARMS, 59% of the patients (n = 215) showed no vulnerability, 21% (n = 76) presented a degree of vulnerability (e.g., state/trait vulnerability, schizotypal personality disorder, −30% of SOFAS score), 13% (n = 47) showed Attenuated Psychotic Syndrome (APS), 1% (n = 4) were classified as Brief Limited Intermittent Psychotic Syndrome (BLIPS), and 6% (n = 22) were diagnosed with psychosis. Considering the three UHR categories included in CAARMS, the percentage of UHR subjects was 35% (n = 127) of the sample.

This sample of young people identified as UHR through the assessment implemented by the 2013 CCM Project was subsequently monitored by mental health service providing evidence-based interventions. The project did not plan modifications of the patterns of care of the local services. No follow-up evaluation of the recruited subjects was programmed, and no action to change psychological or pharmacological treatment or risk assessment was implemented. The local treatment guidelines and clinical experience were followed.

The absence of a control group to compare these data to previous results collected in the Italian mental health systems is a major limit of the present work. The project, however, aimed at implementing best available practices in the field of youth severe mental illness prevention by a strategical change in service provision. Institutional funding objectives did not include determining whether the implemented changes effectively improved the cohort outcome. This crucial question should be answered by further investigations. On the other hand, evidence of successful implementation of the model was simply the realization of the proposed actions ending in the recruitment and care of a relevant cohort of help-seeking youths.

## Future Strategies

The prevention-oriented model with integrated community coalitions proved transferability to Italian services. In fact, the CCM2013 Project integrated in routine mental health services the high-risk paradigm with a public health perspective focused on a wider concept of youth mental health and secondary prevention through the application of the Community Coalition model (19).

According to this perspective, the preventive approach had been implemented at two levels. Inside the mental health services, specific actions to improve clinical assessment of young patients have been promoted. Outside the mental health services, local community coalitions have been developed to raise awareness of youth mental health, to reduce stigma, and to take responsibility for improving communities’ ability to deal with the social problems related to youth mental illness (i.e., school dropout or early school-leaving, social withdrawal). Moreover, low-stigmatized and appropriate locations detached from the main adult mental health services as well as from the child services have been implemented to improve access and help-seeking behaviors in young general population.

The major limit of the CCM2013 Project resides in the lack of assessment of the efficacy of the implementation actions, without providing empirical evidence showing the increased mental health service accessibility for youth. Future research studies should include specific evaluation procedures and outcomes, as well as control groups to verify the efficacy of the integrated multicentric actions aimed at promoting service accessibility in young populations.

However, the above described perspective is in line with the principles of youth service transformation presented by Malla and colleagues (30), aimed at promoting early and simplified access to multidisciplinary services for young people presenting with a wide range of mental health problems. Similarities between Italian CCM2013 Project and other worldwide programs may be highlighted. An example is the Headspace Youth Psychiatry Program, implemented through different centers across Australia, aiming at widening the accessibility of young individuals from 12 to 25 years experiencing mental distress, involving multidisciplinary mental health teams and solid online support programs (31).

Further, the project’s limitations should be acknowledged: first, the temporal stability of the organizational changes adopted and, in particular, of the integration of child–adolescent and adult mental health services; second, the transferability of the model to the whole Italian country, given the relevant regional differences in mental health policies and funding; and third, the project did not contemplate efforts to create strong online communication strategies.

Despite these limitations, some recommendations were further developed to improve youth mental health programs targeting young subjects with serious mental illness in Italy. Several actions can be advised: i) strengthen at a large scale the promotion of community coalitions to encourage the detection of signs and symptoms of mental distress and vulnerability in young subjects; ii) designing actions to facilitate accessibility and attractiveness to services to youth groups in general and with elevated risk for severe mental illness; and iii) creating established integrated child–adult mental health. Future directions should focus also on developing a user-friendly online platform and online support resources to improve youth’s accessibility to mental health prevention strategies.

## Data Availability Statement

The raw data supporting the conclusions of this manuscript will be made available by the authors, without undue reservation, to any qualified researcher.

## Ethics Statement

Local ethic committees’ approval was not required as per the Italian legislation. The project was approved and funded by the National Centre for Disease Prevention and Control and supported by the Lombardy Region. The CCM2013 Project was an implementation project aimed at transferring best clinical practices into routine mental health care. Thus, patients’ data were only gathered for clinical and not for research purposes. Specific written informed consent was obtained from each recruited subject or from his/her parents if under age.

## Author Contributions

All authors listed have made a substantial, direct, and intellectual contribution to the work, and approved it for publication.

## Funding

Research for this paper was supported by “Centro Nazionale per la Prevenzione e il Controllo delle Malattie” (CCM is the Italian acronym for the National Centre for Disease Prevention and Control), Ministero della Salute, Project CCM2013. Project’s title: “Interventi integrati per favorire il riconoscimento e il trattamento precoce dei disturbi psichici gravi in età giovanile (15-24 anni) in gruppi di popolazioni a rischio”.

## Conflict of Interest

The authors declare that the research was conducted in the absence of any commercial or financial relationships that could be construed as a potential conflict of interest.

## References

[1] Yung AR Fusar-Poli P Borgwardt S Bechdolf A Addington J Riecher-Rossler A The psychosis high-risk state. a comprehensive state-of-the-art-review. JAMA Psychiatry (2013) 70(1):107–20. 10.1001/jamapsychiatry.2013.269 PMC4356506 23165428

[2] Ruhrmann S Schultze-Lutter F Salokangas RK Heinimaa M Linszen D Dingemans P Prediction of psychosis in adolescents and young adults at high risk: results from the prospective European prediction of psychosis study. Arch Gen Psychiatry (2010) 67(3):241–51. 10.1001/archgenpsychiatry.2009.206 20194824

[3] Fusar-Poli P Bonoldi I Yung AR Borgwardt S Kempton MJ Valmaggia L Predicting psychosis: meta-analysis of transition outcomes in individuals at high clinical risk. Arch Gen Psychiatry (2012) 69:220–9. 10.1001/archgenpsychiatry.2011.1472 22393215

[4] Schultze-Lutter F Michel C Schmidt SJ Schimmelmann BG Maric NP Salokangas RKR EPA guidance on the early detection of clinical high risk states of psychoses. Eur Psychiatry (2015) 30:405–16. 10.1016/j.eurpsy.2015.01.013 25735810

[5] McGorry PD Hickie IB Yung AR Pantelis C Jackson HJ Clinical staging of psychiatric disorders: a heuristic framework for choosing earlier, safer and more effective interventions. Aust N Z J Psychiatry (2006) 40(8):616–22. 10.1080/j.1440-1614.2006.01860.x 16866756

[6] Van der Gaag M Smit F Bechdolf A French P Linszen DH Yung AR Preventing a first episode of psychosis: meta-analysis of randomized controlled prevention trials of 12 month and longer-term follow-ups. Schizophr Res (2013) 149:56–62. 10.1016/j.schres.2013.07.004 23870806

[7] Yung AR Phillips LJ Nelson B Francey SM Panyuen H Simmons MB Randomized controlled trial of interventions for young people at ultra high risk for psychosis: 6-month analysis. J Clin Psychiatry (2011) 72:430–40. 10.4088/JCP.08m04979ora 21034687

[8] Klosterkötter J Shultze-Lutter F Bechdolf A Ruhrmann S Prediction and prevention of schizophrenia: what has been achieved and where to go next?. World Psychiatry (2011) 10:165–74. 10.1002/j.2051-5545.2011.tb00044.x PMC3190243 21991266

[9] vanOs J Guloksuz S A critique of the “ultra-high risk” and “transition”paradigm. World Psychiatry (2017) 16:200–6. 10.1002/wps.20423 PMC5428198 28498576

[10] McGorry PP Killackey E Yung A Early intervention in psychosis: concepts, evidence and future directions. World Psychiatry (2008) 7:148–56. 10.1002/j.2051-5545.2008.tb00182.x PMC2559918 18836582

[11] Rutigliano G Valmaggia L Landi P Frascarelli M Cappucciati M Persistence or recurrence of non-psychotic comorbid mental disorders associated with 6-year poor functional outcomes in patients at ultra high risk for psychosis. J Affect Disord (2016) 203:101–10. 10.1016/j.jad.2016.05.053 27285723

[12] Lin A Wood SJ Nelson B Beavan A McGorry P Yung, AR Outcomes of nontransitioned cases in a sample at ultra-high risk for psychosis. Am J Psychiatry (2015) 172(3):249–58. 10.1176/appi.ajp.2014.13030418 25727537

[13] Linscott RJ van Os J An updated and conservative systematic review and meta-analysis of epidemiological evidence on psychotic experiences in children and adults: on the pathway from proneness to persistence to dimensional expression across mental disorders. Psychol Med (2013) 43(6):1133–49. 10.1017/S0033291712001626 22850401

[14] Kelleher I Keeley H Corcoran P Lynch F Fitzpatrick C Devlin N Clinicopathological significance of psychotic experiences in non-psychotic young people: evidence from four population-based studies. Br J Psychiatry (2012) 201:26–32. 10.1192/bjp.bp.111.101543 22500011

[15] Cocchi A Cavicchini A Collavo M Ghio L Macchi S Meneghelli A Implementation and development of early intervention in psychosis services in Italy: a national survey promoted by the associazione italiana interventi precoci nelle psicosi. Early Interv Psychiatry (2018) 12(1):37–44. 10.1111/eip.12277 26416725

[16] Masillo A Brandizzi M Nelson B Lo Cascio N Saba R Youth mental health services in Italy: an achievable dream?. Early Interv Psychiatry (2018), Jun, 12 (3), 433–43. 10.1111/eip.12328 27061589

[17] Lora A Barbato A Cerati G Erlicher A Percudani M The mental health system in Lombardy, Italy: access to services and patterns of care. Soc Psychiatry Psychiatr Epidemiol (2011) 47:447–54. 10.1007/s00127-011-0352-1 21293841

[18] Corrao G Soranna D Merlino L Monzani E Vigano C Lora A Do patterns of mental healthcare predict treatment failure in young people with schizophrenia? Evidence from an Italian population-based cohort study. BMJ Open (2015) 5:e007140. 10.1136/bmjopen-2014-007140 PMC4458586 26041489

[19] Butterfoss FD Kegler MC Emerging theories in health promotion practice and research. In: DiClemente RJ Crosby RA Kegler MCeditors. Toward a comprehensive understanding of community coalitions: moving from practice to theory. Jossey-Bass (2002). 157–93.

[20] Hawkins JD Oesterle S Brown EC Arthur MW Abbott RD Fagan AA Results of a type 2 translational research trial to prevent adolescent drug use and delinquency: a test of ommunities that care. Arch Pediatr Adolesc Med (2009) 163:789–98. 10.1001/archpediatrics.2009.141 PMC2740999 19736331

[21] Gloppen KM Arthur MW Hawkins JD Shapiro VB Sustainability of the communities that care prevention system by coalitions participating in the Community Youth Development Study. J Adolesc Health (2012) 51(3):259–64. 10.1016/j.jadohealth.2011.12.018 PMC3428591 22921136

[22] Percudani M Parabiaghi A D’Avanzo B Bassi M Cardamone G Costantino A Un modello di prevenzione e cura dei disturbi psichici gravi in eta’ giovanile (15-24 anni). Psichiatria Oggi (2016) XXIX2, 54–65.

[23] Piccinelli M Bisoffi G Bon MG Cunico L e Tansella M Validity and test–retest reliability of the Italian version of the 12-item general health questionnaire in general practice, a comparison between three scoring methods. Compr Psychiatry (1993) 34:198–205. 10.1016/0010-440X(93)90048-9 8339539

[24] Azzali S Pelizza L Paterlini F Garlassi S Scazza I Reliability of the italian version of the 16-item prodromal questionnaire (iPQ-16) for psychosis risk screening in a young help-seeking community sample. J Psychopathol (2018) 24:16–23. 10.1016/j.schres.2018.03.023

[25] Ising HK Veling W Loewy R Rietveld M Rietdijk J Dragt S The Validity of the 16-item version of the prodromal questionnaire (PQ-16) to screen for ultra high risk of developing psychosis in the general help-seeking population. Schizophr Bull (2012) 38(6):1288–96. 10.1093/schbul/sbs068 PMC3713086 22516147

[26] Yung AR Yuen HP McGorry PD Kelly D Dell’Olio M Francey SM Mapping the onset of psychosis: the comprehensive assessment of at- risk mental states. Austral N Z J Psychiatry (2005) 39(11-12):964–71. 10.1080/j.1440-1614.2005.01714.x 16343296

[27] Diagnostic and Statistical Manual of Mental Disorders, 4th ed. *(DSM-IV)* Washington: APA (1994).

[28] Goldman HH Skodol AE Lave TR Revising axis V for DSM-IV: a review of measures of social functioning. Am J Psychiatry (1992) 149(9):1148–56. 10.1176/ajp.149.9.1148 1386964

[29] Wing JK Beevor AS Curtis RH Park SGB Hadden J Burns A Health of the nation outcome scales (HoNOS): research and development, 1998. Br J Psychiatry (1998) 172pp(1):11–8. 10.1192/bjp.172.1.11 9534825

[30] Malla A Shah J Iyer S Boksa P Joober R Andersson N Youth mental Health should be a top priority for health care in canada. Can J Psychiatry (2018) 63(4):216–22. 10.1177/0706743718758968 PMC5894919 29528719

[31] Bassilios B Telford N Rickwood D Spittal MJ Pirkis J Complementary primary mental health programs for young people in Australia: access to allied psychological services (ATAPS) and headspace. Int J Ment Health Syst (2017) 11:19. 10.1186/s13033-017-0125-7 28203274 PMC5301357

